# Reliability, validity and responsiveness of the Chinese version of the Rotator Cuff Quality of Life Index (RC-QOL) in patients with rotator cuff disorders

**DOI:** 10.1371/journal.pone.0206347

**Published:** 2018-11-08

**Authors:** Wei Wang, Chen Zhang, Lin Cui, Qing-yun Xie, Zhen-yu Jia, Wei Zheng

**Affiliations:** 1 Department of Orthopedics, Chengdu Military General Hospital, Chengdu city, People's Republic of China; 2 Department of Orthopedics, Changhai Hospital, Shanghai, People's Republic of China; Public Library of Science, UNITED KINGDOM

## Abstract

**Background:**

The Rotator Cuff Quality of Life Index (RC-QOL) is a scale designed to evaluate the impact of rotator cuff (RC) disorders on the general quality of life of patients. Our study aims to adapt the RC-QOL into Chinese and to assess its reliability, validity and responsiveness in Chinese patients with RC disorders.

**Methods:**

First, we developed the Chinese version of RC-QOL (C-RC-QOL) through a five-step procedure. Next, the recruited patients gave three rounds of responses to the C-RC-QOL, Medical Outcomes Study Short Form 36 (SF-36) and Oxford Shoulder Score scales (OSS). Then, we calculated the Cronbach’s alpha, standard error of measurement (SEM), minimally detectable change (MDC), intra-class correlation coefficient (ICC), Pearson's correlation coefficient (*r*), effect size (ES) and standardized response mean (SRM) to evaluate the reliability, validity and responsiveness of the C-RC-QOL respectively. The unidimensionality of each subscale was assessed by principal component analysis (PCA) of the residuals.

**Results:**

Overall, 124 patients with RC disorders successfully completed the first two rounds of the scales, and 108 patients completed the last round of the scales. Good or excellent internal consistency (Cronbach’s alpha = 0.953) was found in the overall scale and subscales of the C-RC-QOL, and good or excellent test-retest reliability (ICC = 0.854) was found as well. The SEM and MDC values of the C-RC-QOL were 4.6 and 12.8 respectively. Moderate, good or excellent correlations (*r =* 0.452–0.839) were obtained between the physical subscales of the C-RC-QOL and the OSS, as well as the physical subscales of the SF-36; similar results were obtained between the emotion subscale of the C-RC-QOL and the mental subscales of the SF-36 (*r =* 0.490–0.733), which, illustrated the good validity of the C-RC-QOL. In addition, high responsiveness was observed in the overall scale and subscales of the C-RC-QOL (ES = 1.77, SRM = 1.98). The unidimensionality of five subscales was respected according to PCA of the residuals.

**Conclusions:**

The C-RC-QOL scale is reliable, valid and responsive for the evaluation of Chinese-speaking patients with RC disorders and it would be an effective instrument.

## 1. Introduction

Shoulder pain is the third most common musculoskeletal symptom encountered in medical practice after back and neck pain [1], and rotator cuff (RC) disorders are the most common cause of shoulder pain, accounting for 35–45% of rendered diagnoses [2]. The major symptoms of RC disorders include pain with motion, night pain and stiffness [3]. In addition, some studies have confirmed that RC disorders are associated with some dysmetabolic diseases [4]. The functional losses cause negative impacts on the patient’s activities of daily living, work status and sporting activities, consequently influencing health-related quality of life (HRQOL) [5,6].

More and more studies have been dedicated to developing patient reported outcome measures (PROMs) to assess the health-related quality of life of patients since the 1980s [7]. Generally, by asking patients to complete these scales independently, researchers are able to obtain information about the severity of the patients’ condition, and then determine more appropriate treatments [8]. PROMs can be divided into generic and disease-specific measures based on their primary purpose. Generic scales, such as the Medical Outcomes Study Short Form 36 (SF-36), are used to evaluate the general status of patients, while disease-specific scales are used to assess specific patient groups; examples of disease-specific scales include Lysholm knee score for anterior cruciate ligament injury [9], the Western Ontario Osteoarthritis of the Shoulder Index (WOOS) for shoulder osteoarthritis [10], and the Rotator Cuff Quality of Life Index (RC-QOL) [11] and Western Ontario Rotator Cuff Index (WORC) [12] for RC disorders.

Because of the increasing number of large multicenter international studies and the requirement for globally meaningful epidemiological and/or therapeutic study results, there is a need for cross-cultural adaptation and validation of PROMs [13]. When one reliable, valid scale is being used in populations with different cultures, it is necessary to test the psychometric properties of the scale rather than simply translating the content in order to avoid evaluation error caused by cultural differences [14,15].

At present, only two scales addressing shoulder joint-related diseases, the Disability of Arm, Shoulder and Hand (DASH) and Oxford Shoulder Score (OSS) measures, have been translated and cross-culturally adapted into Chinese [16,17]. However, these two scales are not specifically designed for RC disorders. The DASH is for patients with upper extremity musculoskeletal conditions and the OSS for patients having shoulder operations other than stabilization [18]. Although a recent systematic review confirmed that upper limb shoulder specific scales are not less responsive or valid than condition specific scales for RC disorders [19], there is value in the use of a specific scale for a particular condition, where such scale is available.

The RC-QOL is a simple disease-specific questionnaire that was developed in the 1990s to evaluate the impact of RC disorders on health-related quality of life [20]. Initially, relevant items were generated from a thorough review of the literature, from discussions with clinicians experienced in the area of RC disorders and from "patients with a full spectrum of rotator cuff disease" [11]. In 2000, Hollinshead et al. eliminated 21 items that were redundant, unimportant or had poor taste-retest reliability reducing the number of items from 55 to 34. The final 34-item questionnaire has been tested and showed to have high reliability, validity and responsiveness, as well as an ability to discriminate between large and massive cuff tears [11]. The original version of the RC-QOL was created in English and has been translated and validated into German, Turkish and Italian [13,20,21]. Unfortunately, no Chinese version has been published yet even though China has the largest population of patients with RC disorders.

Therefore, we aimed to translate and adapt the RC-QOL into a Chinese version (C-RC-QOL) and evaluated the reliability, validity and responsiveness of the C-RC-QOL in a cohort of native Chinese-speaking patients with RC disorders.

## 2. Patients and methods

### 2.1 Translation and cross-cultural adaptation

The guidelines in the related published guide were followed in translating the original version of the RC-QOL scale [22,23]. The translation process consisted of five steps. 1) Forward translation: the forward translation from English to Chinese was completed separately by two bilingual translators who were native speakers of Chinese; one of the translators was an orthopedist and the other was a specialized translator with no medical background. 2) Translation integration: a meeting was held to construct the first version of the C-RC-QOL by combining the translation of the two translators. 3) Backward translation: two additional translators, who were native speakers of English and had never read the English version of the C-RC-QOL, separately translated the first version of the C-RC-QOL back into English. 4) Prefinal version: a meeting was held to discuss any discrepancies, ambiguities or other expression issues. Considering all the suggestions, we developed the prefinal version of the C-RC-QOL. 5) Final version of C-RC-QOL: twenty patients with RC disorders were invited to complete the prefinal version of C-RC-QOL. Their feedback was collected and a third consensus meeting was held to construct the final version of the C-RC-QOL.

### 2.2 Patients and data collection

The subjects of this study were recruited among patients with RC disorders who visited Chengdu Military General Hospital and Shanghai Changhai Hospital from January 2015 to March 2016. The criteria used in our recruitment were as follows: at least 18 years of age; native speakers of Chinese, able to complete the scale independently, diagnosed with RC disorders and prepared to receive shoulder arthroscopic surgery. The initial diagnoses of the RC patients were performed independently by two orthopaedic attending physicians (the first authors, WW and CZ). The diagnoses were mainly based on the patients’ medical history (shoulder joint injury or overuse) and signs: 1) shoulder joint pain and aggravation during abduction, 2) rotator cuff muscle strength decreased, 3) typical signs positive (Job sign, Lift off sign and Hornblower's sign). Patients who were initially diagnosed with RC disorders would be assessed by the same orthopedic chief physician (the corresponding author, WZ) and then undergo shoulder MRI to further confirm the diagnoses. And we adhered strictly to the indications for shoulder arthroscopic surgery [24]. Moreover, we excluded patients with cervical spine damage (history of neck injury accompanied by neck and shoulder symptoms, or previously diagnosed with cervical spondylosis or herniation of cervical disc), patients with upper limb injuries affecting the shoulder function, and patients with systemic diseases such as diabetes or high blood pressure. Patients who received conservative treatment before arthroscopic surgery were also excluded to avoid affecting the evaluation of the test-retest reliability. The number of included subjects met the sample size requirement for health scale studies proposed by Terwee, et al [25], which requires that there be results from at least 100 subjects for internal consistency analysis and results from at least 50 subjects to assess floor or ceiling effects, reliability, and validity. All subjects carefully read and signed the Informed Consent Form, and this study was approved by the Ethics Committee of Chengdu Military General Hospital (Permit Number: 2015–0103).

The patients were asked to provide demographic information such as sex, age and weight on the first day of enrollment and to independently complete four scales including the C-RC-QOL, OSS, SF-36 and C-WORC (for another study) in a quiet meeting room; Wei Wang and Chen Zhang (the first authors) were responsible for distributing and collecting the patient questionnaires. One week later, which was also the day before the arthroscopic surgery, the patients completed the C-RC-QOL for the second time to evaluate its test-retest reliability. Six months after surgery, when the patients came to our hospital for regular check-ups, they completed the C-RC-QOL for the third time to help evaluate its responsiveness.

### 2.3 Scales

The RC-QOL is a self-assessment scale that contains 34 items representing five subscales: symptoms and physical complaints (16 items), work-related concerns (4 items), sports and recreation (4 items), lifestyle (5 items), and social and emotional aspects (5 items), which encompass all aspects of heath as defined by the World Health Organization [26]. Each item is answered on a 100-mm visual analog scale: a score of 0 implies the worst condition, and a score of 100 indicates the best possible PROM. The total score is then represented as a percentage [11].

The OSS is a self-assessment scale that has been developed for patients with shoulder pain. It evaluates the effects of shoulder joint disease on patients’ daily life functioning and quality of life using 12 items [27]. Each item has five response options scored from 0 to 4, and therefore the total score ranges from 0 (worst) to 48 (best). A lower score indicates that the status of shoulder joint is worse [28]. The SF-36 is a generic quality of life scale that consists of eight subscales. It is used to assess the mental health of patients, physical functioning and social role functioning. Each section has its own scoring method and is transformed into a 0–100 scale; a lower score indicates worse quality of life and severer disability [29]. The two scales mentioned above have been translated into Chinese, and their Chinese versions have been demonstrated to have good reliability, validity and responsiveness [17,30].

### 2.4 Psychometric assessments and statistical analysis

To validate the reliability of the C-RC-QOL, we tested its internal consistency and test-retest reliability. Cronbach’s alpha was used to indicate the scale’s internal consistency. A scale is deemed to show acceptable, good or excellent internal consistency at a Cronbach coefficient of 0.7, 0.8 or 0.9 respectively [24]. The scale’s test-retest reliability was evaluated by comparing the outcomes obtained from the first two rounds of the C-RC-QOL. The intraclass correlation coefficient (ICC) derived from a two-way analysis of variance in a random effect model was used here as the indicator. A scale is considered to have good or excellent test-retest reliability when the ICC score is higher than 0.8 or 0.9 respectively [31]. We also calculated the standard error of measurement (SEM) and minimal detectable change (MDC) to evaluate the reliability of the instrument. SEM and MDC were calculated with the following formulas: SEM = SD×√(1-ICC), and MDC = SEM×1.96×√2, where SD is the standard deviation of the first test scores [32]. Furthermore, we created Bland-Altman plots to search for systematic error between the investigations [33].

Next, we assessed the content validity and construct validity of C-RC-QOL. Aspects of content validity include the comprehensiveness and relevance of items [34]. It is believed that a scale has good comprehensiveness when each item’s response rate > 95%, each subscale’s ceiling/floor effects < 15% and no subject reports difficulty in comprehension while completing the scale [25,35]. In addition, a rehabilitation medicine expert and three orthopedic specialists were invited to judge whether the items were relevant for the construct to be measured and for patients with RC disorders [34]. As there is no gold standard for evaluating the validity of the C-RC-QOL, we assessed its construct validity through hypothesis testing [34]. The OSS and SF-36 were used as the contrast scales. Based on the contents of each scale, we hypothesized that the physical subscales of C-RC-QOL (symptoms and physical complaints, work-related concerns, sports and recreation, lifestyle) should be well correlated (*r* = 0.4–1.0) with the physical subscales of SF-36 (physical functioning, role-physical, bodily pain, general health) and fairly of poorly correlated (*r* = 0–0.4) with the mental subscales of SF-36 (vitality, social functioning, role-emotional, mental health). Correspondingly, the emotion subscales of C-RC-QOL (social and emotional aspects) should be well correlated (*r* = 0.4–1.0) with the mental subscales of SF-36 and not well correlated (*r* = 0–0.4) with the OSS and physical subscales of SF-36. Further, considering that the OSS is a shoulder-specific scale and the SF-36 is a generic scale, we hypothesized that C-RC-QOL should be more correlated with OSS than with SF-36. Based on the above hypotheses (listed in Table 1), we calculated the correlations among the subscales of C-RC-QOL, SF-36 and OSS using Pearson’s correlation coefficient (*r*) as an indicator; and then compared these results with our hypotheses to assess the construct validity of C-RC-QOL. The correlations were judged as poor (*r* = 0–0.2), fair (*r* = 0.2–0.4), moderate (*r* = 0.4–0.6), good (*r* = 0.6–0.8), or excellent (*r* = 0.8–1.0) [36].

**Table 1:** Hypotheses for the correlations between the subscales of the C-RC-QOL, SF-36 and OSS.

Pearson correlation coefficient (*r*)	Subscales of C-RC-QOL				
	Symptoms and physical	Work-related concerns	Sports and recreation	Lifestyle	Social and emotions
**OSS**	*r* > 0.6	*r* > 0.6	*r* > 0.6	*r* > 0.6	*r* < 0.4
**SF-36**					
Physical function	*r* > 0.4	*r* > 0.4	*r* > 0.4	*r* > 0.4	*r* < 0.4
Role physical	*r* > 0.4	*r* > 0.4	*r* > 0.4	*r* > 0.4	*r* < 0.4
Bodily pain	*r* > 0.4	*r* > 0.4	*r* > 0.4	*r* > 0.4	*r* < 0.4
General health	*r* > 0.4	*r* > 0.4	*r* > 0.4	*r* > 0.4	*r* < 0.4
Vitality	*r* < 0.4	*r* < 0.4	*r* < 0.4	*r* < 0.4	*r* > 0.4
Social function	*r* < 0.4	*r* < 0.4	*r* < 0.4	*r* < 0.4	*r* > 0.4
Role emotional	*r* < 0.4	*r* < 0.4	*r* < 0.4	*r* < 0.4	*r* > 0.4
Mental health	*r* < 0.4	*r* < 0.4	*r* < 0.4	*r* < 0.4	*r* > 0.4

C-RC-QOL: Chinese version of the Rotator Cuff Quality of Life scale; OSS: Shoulder Oxford score;

We evaluated the responsiveness of C-RC-QOL by comparing the scale results before and 6 months after arthroscopic treatment. Standardized response mean (SRM) and effect size (ES) were the two indices used to evaluate responsiveness. SRM was defined as the mean change between the two time points divided by the standard deviation (SD) of this change. ES was defined as the mean change between pretreatment results and 6-month post-treatment results divided by the SD of the pretreatment C-RC-QOL score [37]. ES and SRM were considered large if greater than 0.80, moderate if between 0.51 and 0.80 and small if lower than 0.50 [38].

The unidimensionality of the five subscales was assessed by principal component analysis (PCA) of the residuals [39]. Common criteria for unidimensionality are that at least 50% of the variance should be explained by the first dimension (i.e. the lifestyle subscale) and that the eigenvalue for the second largest dimension should be < 2. An eigenvalue of 2 or greater indicates that a second dimension might be present [40].

Statistical analysis was performed using the programs SPSS (version 20.0) and WINSTEPS Rasch (version 3.72.3). *P* value of 0.05 or less were considered statistically significant.

## 3. Results

### 3.1 Patients

Among the patients with RC disorders visiting Chengdu Military General Hospital and Shanghai Changhai Hospital from January 2015 to March 2016, 152 of them (74 male and 78 female) met our criteria and were invited to participate in our study. In the end, 28 patients explicitly refused to participate in this study, and 124 patients (81.6% of those invited, 69 male and 55 female) became our subjects. All subjects completed the first two rounds of responses. However, 16 subjects failed to finish the third round due to loss to follow-up (6 months after their shoulder arthroscopic surgery). By reviewing the medical records of patients who were lost to follow-up, they did not have specific or different aspects compared to other patients. Consequently, the results from 124 subjects were included in the reliability and validity of C-RC-QOL tests, while only the results of 108 subjects were assessed in the responsiveness test. The detailed demographic information of participants was listed in Table 2.

**Table 2:** Demographic and clinical characteristics of participants.

Characteristics	Number (%) or Mean ± SD
Age (years)	47.3 ± 9.5
Range	20–66
Age groups	
≦30	8 (6.5%)
31–45	40 (32.3%)
46–60	64 (51.6%)
≧61	12 (9.7%)
Gender	
Female	55 (44.4%)
male	69 (55.6%)
Affected side	
Right	64 (51.6%)
Left	60 (48.4%)
Dominant side	
Dominant	71 (57.3%)
Nondominant	53 (42.7%)
Pain duration (months)	30.7 ± 20.2
BMI (Kg/m^2^)	23.7 ± 4.6

BMI: body mass index

### 3.2 Translation and cross-cultural adaptation process

Both forward and backward translations of RC-QOL were completed successfully. For our localized translation, the description “cutting food for preparation or at meals” in item 7 was adapted to “using both hands to hold a bowl and chopsticks when eating a meal”, and the description “driving a motor vehicle” in item 13 was adapted to “driving a motor vehicle or riding a bicycle”. Twenty patients (10 male and 10 female) with RC disorders completed the prefinal version of C-RC-QOL, and no participant complained of irregularities in the questions or difficulties in understanding the questions. This version was used as final version in the subsequent validation phase without any further change.

### 3.3 Reliability

**Fig 1:**
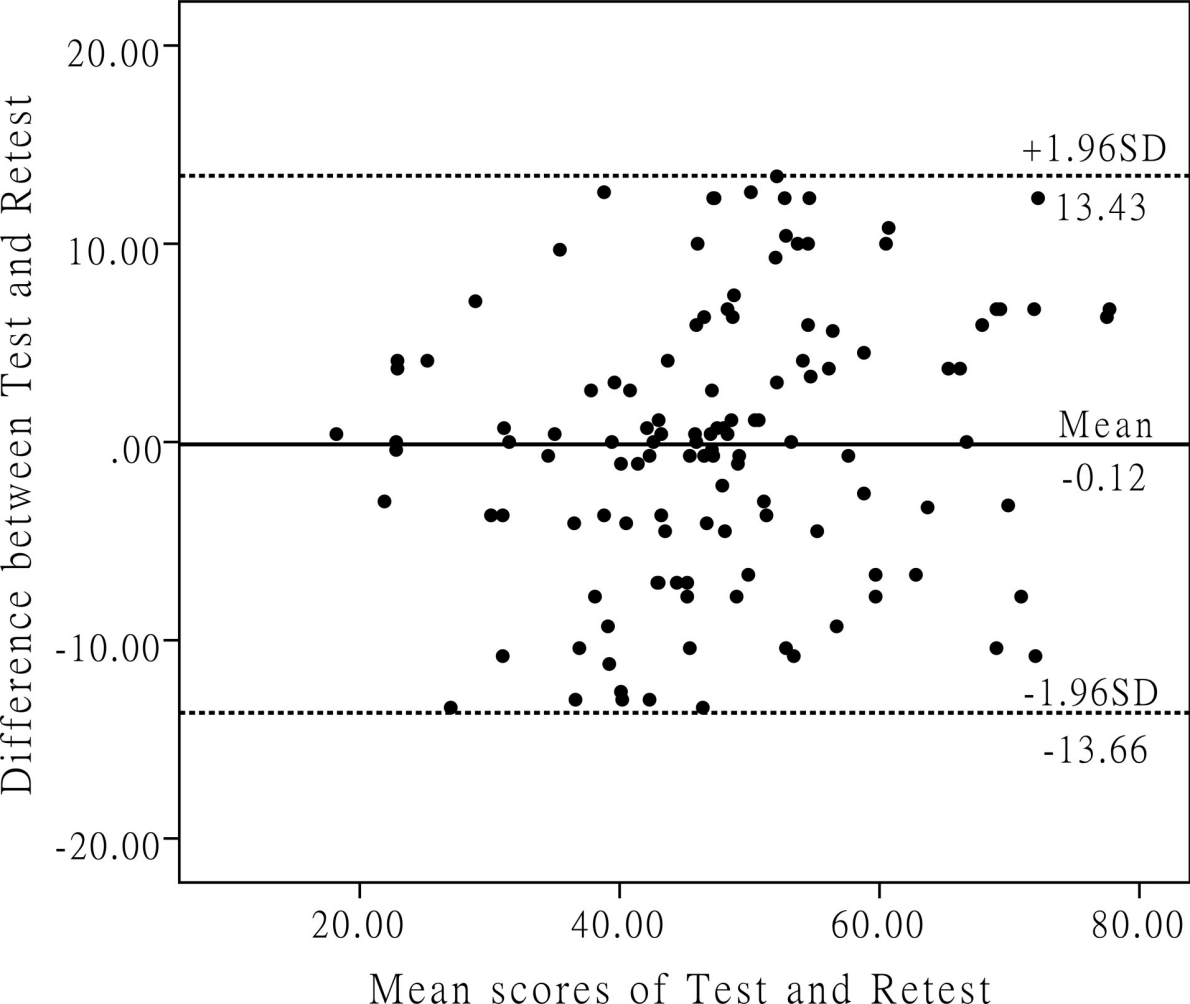
This figure shows the Bland-Altman plots of the test-retest reliability of the C-RC-QOL. Each data point indicates how the difference between the two test sessions for an individual patient compares to the mean of the two sessions for scores of each C-RC-QOL. The interval between the two sessions was 1 week. The dashed line shows the 95% (± 1.96 SD) limits of agreement.

The overall scale of the C-RC-QOL, with a Cronbach’s alpha of 0.953, had excellent internal consistency. In addition, each of its subscales with Cronbach’s alpha values ranging from 0.806 to 0.942, had good or excellent internal consistency (Table 3). The overall test-retest reliability of the C-RC-QOL was good with an ICC value of 0.854, and each subscale’s test-retest reliability was good or excellent, with ICC values ranging from 0.824 to 0.954 (Table 4). The Bland-Altman plots revealed that the test-retest differences for the C-RC-QOL were centered around zero (Fig 1), which indicated that there was no systematic error in the data obtained from the first two rounds of completed scales and confirmed the good test-retest agreement of the C-RC-QOL. The SEM was 4.6 for the overall scale and 3.5–6.8 for the subscales, and the MDC was 12.8 for the overall scale and 9.6–19.0 for the subscales (Table 4).

**Table 3:** Distribution and internal consistency for the subscales of the C-RC-QOL.

Subscale	Mean ± SD	Observed range	Theoretical range	Missing items n (%)^a^	Floor effect (%)^b^	Ceiling effect (%)^b^	Cronbach's alpha
Overall scale	47.8 ± 12.1	18.0–77.4	0–100	4 (3.2)	0	0	0.953
Symptoms and physical	47.4 ± 13.9	8.4–87.2	0–100	4 (3.2)	0	0	0.942
Work-related concerns	49.0 ± 15.2	6.5–87.0	0–100	1 (0.8)	0	0	0.827
Sports and recreation	47.0 ± 15.1	6.0–89.3	0–100	0 (0)	0	0	0.806
Lifestyle	47.4 ± 16.1	0–90.6	0–100	0 (0)	0.8	0	0.863
Social and emotions	48.9 ± 16.3	0–90.8	0–100	0 (0)	1.6	0	0.851

C-RC-QOL: Chinese version of the Rotator Cuff Quality of Life scale; SD: Standard deviation.

^a ^Number of patients with some missing items in the subscale or overall scale.

^b^ Percentage of patients with the worst (floor effect) and the best (ceiling effect) score

**Table 4:** Reliability, responsiveness and results of the PCA of residuals for the C-RC-QOL ^a^.

Subscale	ICC (CI range)	ES (CI range)	SRM (CI range)	SEM (CI range)	MDC	Variance explained by measures (%)	Eigenvalue
Overall scale	0.854 (0.798–0.895)	1.77 (1.56–1.96)	1.98 (1.65–2.16)	4.6 (4.2–5.1)	12.8	-	-
Symptoms and physical	0.882 (0.836–0.916)	1.74 (1.61–1.92)	1.78 (1.56–1.96)	4.8 (4.3–5.4)	13.2	51.3	1.8
Work-related concerns	0.934 (0.907–0.953)	1.29 (1.13–1.38)	1.18 (1.02–1.34)	3.9 (3.4–4.5)	10.8	60.7	1.0
Sports and recreation	0.919 (0.887–0.943)	1.93 (1.83–2.08)	1.84 (1.59–2.05	4.3 (4.0–4.7)	11.9	56.4	1.3
Lifestyle	0.954 (0.935–0.968)	2.04 (1.95–2.18)	1.83 (1.56–1.96)	3.5 (3.1–4.0)	9.6	55.1	1.4
Social and emotions	0.824 (0.759–0.874)	2.03 (1.88–2.26)	1.70 (1.48–1.91)	6.8 (6.2–7.6)	19.0	58.6	1.1

C-RC-QOL: Chinese version of the Rotator Cuff Quality of Life scale; PCA: principal components analysis; ICC: intra-class correlation coefficient; ES: effect size; SRM: standardized response mean; CI: 95% confidence interval; SEM: standard error of measurement; MDC: minimally detectable change.

^a^ The sample size for the analysis of reliability and PCA was 124, and the sample size for the analysis of responsiveness was 108.

### 3.4 Validity

More detailed examination found that there were four subjects who did not respond to one item of the symptoms and physical subscale (4/124, 3.2%) and one subject who did not respond to one item of the work-related subscale (1/124, 0.8%) (Table 3). The score distribution showed that there was no ceiling effect (0%) or floor effect (0–1.6%) in the overall C-RC-QOL scale and any of the subscales. In addition, no subjects reported that any content in questionnaire was hard to understand after completing the C-RC-QOL. After their assessment, the invited rehabilitation medicine expert and orthopedic specialists affirmed that the data acquired from the C-RC-QOL were adequate to evaluate the HRQOL of patients with RC disorders, and they did not recommend adding or deleting any item. In summary, we concluded that the C-RC-QOL had a good content validity.

Construct validity assessment data are listed in Table 5. The physical subscales of C-RC-QOL were at least moderately correlated with the OSS and physical subscales of SF-36 (*r* = 0.452–0.839), and poorly or fairly correlated with the mental subscales of SF-36 (*r* = 0.136–0.309). The emotion subscale of C-RC-QOL was at least moderately correlated with the mental subscales of SF-36 (*r* = 0.490–0.733), and poorly or fairly correlated with the OSS and physical subscales of SF-36 (*r* = 0.014–0.070). In addition, the correlation between the physical subscales of C-RC-QOL and OSS (*r* = 0.630–0.839) were stronger than that between the physical subscales of C-RC-QOL and physical subscales of SF-36 (*r* = 0.452–0.745). The above results were consistent with our hypothesis, indicating the good construct validity of C-RC-QOL.

**Table 5:** Construct validity of the C-RC-QOL.

Pearson correlation coefficient (*P* value) ^a^^,^^b^	Symptoms and physical	Work-related concerns	Sports and recreation	Lifestyle	Social and emotions	Overall scale
**OSS**	0.839 (<0.001)	0.695 (<0.001)	0.735 (<0.001)	0.630 (<0.001)	0.017 (0.848)	0.791 (<0.001)
**SF-36**						
Physical function	0.652 (<0.001)	0.635 (<0.001)	0.603 (<0.001)	0.484 (<0.001)	0.014 (0.880)	0.633 (<0.001)
Role physical	0.638 (<0.001)	0.600 (<0.001)	0.584 (<0.001)	0.505 (<0.001)	-0.023 (0.800)	0.614 (<0.001)
Bodily pain	0.745 (<0.001)	0.679 (<0.001)	0.673 (<0.001)	0.529 (<0.001)	0.019 (0.835)	0.709 (<0.001)
General health	0.572 (<0.001)	0.479 (<0.001)	0.465 (<0.001)	0.452 (<0.001)	0.070 (0.440)	0.551 (<0.001)
Vitality	0.223 (0.013)	0.206 (0.021)	0.235 (0.009)	0.136 (0.131)	0.554 (<0.001)	0.322 (<0.001)
Social function	0.276 (0.002)	0.231 (0.010)	0.264 (0.003)	0.157 (0.081)	0.500 (<0.001)	0.325 (<0.001)
Role emotional	0.294 (0.001)	0.309 (<0.001)	0.294 (0.001)	0.264 (0.003)	0.490 (<0.001)	0.396 (<0.001)
Mental health	0.297 (0.001)	0.301 (0.001)	0.294 (0.001)	0.207 (0.021)	0.733 (<0.001)	0.434 (<0.001)

C-RC-QOL: Chinese version of the Rotator Cuff Quality of Life scale; OSS: Shoulder Oxford score;

^a^ Calculated by the Pearson correlation coefficient (*r*) of the C-RC-QOL with OSS and SF-36.

^b^ The sample size for the analysis of construct validity was 124.

### 3.5. Responsiveness

We evaluated the responsiveness of the C-RC-QOL by comparing the scale completed before and after arthroscopic treatment. Relevant data were listed in Table 3. In general, the average scores of the overall scale and the subscales all increased after the surgery. The ES (1.29–2.04) and SRM (1.18–1.98) values both exceeded 1.00, suggesting that the C-RC-QOL had good responsiveness.

### 3.6 Unidimensionality

The five subscales of the C-RC-QOL all had more than 50% of the variance explained by the measures (51.3%-60.7%) and an eigenvalue of 1.8 or lower (Table 4), suggesting that the unidimensionality of all of the subscales was maintained.

## 4. Discussion

PROMs scales, as important instruments in clinical research and clinical work, not only enable researchers to quantify the functioning status of patients and compare obtained data with findings in other studies, but also allow clinicians to evaluate the condition of their patients. Clinical research is developing rapidly in China, with a large number of papers published each year [41]. Effective scales are greatly needed to support these clinical studies; for example, randomized controlled trials in evidence-based medicine require scales and tests with verified properties [42]. To date, there has been no disease-specific scale available in China that can be used to evaluate patients with RC disorders, a common problem that imposes a considerable burden on the affected person and society. The RC-QOL is a widely used scale to evaluate the functioning status of patients with RC disorders; it has been translated into multiple languages and its reliability, validity and responsiveness have been well established [11, 13, 20, 21, 43]. It is meaningful to translate the RC-QOL into Chinese to serve the largest population in the world.

However, we must note the limitations of our study before proceeding with the discussion of our research results. First, the sample was limited in size and might not fully represent the Chinese population. Second, our translated version of the RC-QOL is in simplified Chinese. While Mandarin is the official language in China, the many minority groups living here have their own languages. Such ethnic and cultural diversity must be taken into consideration in further use. Third, the C-RC-QOL was not used to assess the patients with RC disorders who had received conservative treatment for their RC disorders. This should be dealt with in further research. Finally, a Global Rating of Change Questionnaire was not used for the calculation of ICC/MDC (only stable participants) and ES/SRM (only improved participants), which could have affected the results

In this study, the process of translation and cross-cultural adaptation was successful. Specifically, items 7 and 13 were modified slightly to adapt to the cultural background. As chopsticks, not knife and fork, are the main utensils used by the Chinese to eat food, we replaced the utensils in item 7 with chopsticks and described actions with them accordingly. Further, as bicycles are a common type of vehicle in China, we added them to item 13. No subject reported any problems understanding the questionnaire in our pretest or our formal study.

The C-RC-QOL, including its overall scale and all subscales, was validated with good or excellent internal consistency (Cronbach’s alpha = 0.806–0.953), which is consistent with the results of other cross-culture adaptation studies (Cronbach’s alpha = 0.83–0.98) [13, 20, 21]. Cronbach’s alpha is considered an adequate measure of internal consistency. A low Cronbach’s alpha indicates a lack of correlation between the items in a scale, which makes it unjustified to summarize the items with a single score. A very high Cronbach’s alpha indicates high correlations among the items in the scale (i.e., redundancy of one or more items). Furthermore, a very high Cronbach’s alpha is usually found for scales with a large number of items, because Cronbach’s alpha is dependent upon the number of items in a scale. In general, Cronbach's alpha should not exceed 0.95 [25]. In this study, the Cronbach's alpha of the overall scale was 0.953, suggesting that there were some redundant items, which may also be related to the high number of items in the overall scale (34 items).

The overall scale and all subscales of the C-RC-QOL were validated with good or excellent test-retest reliability (ICC = 0.824–0.954), and this result was also consistent with other similar studies (ICC = 0.77–0.97) [13,20,43]. Specifically, the lifestyle subscale demonstrated the highest ICC (0.954), in part because the daily lives of patients probably did not change much in one week. One week should be an appropriate interval for assessing the scale’s test-retest reliability. It is long enough for patients to forget the responses they provided before, and it is not long enough for patients’ functioning status or lifestyle to change significantly. In addition, subjects were waiting for their shoulder arthroscopic surgery in this week, and therefore would not receive other treatment during this period, which prevents any related errors. We also calculated the SEM and MDC values of the C-RC-QOL, and the MDC has not been calculated in similar studies. SEM provides an estimate of how reliably a scale estimates an individual’s “true score” (i.e., the score that the individuals would obtain if the instrument measured without error) [44]; the SEM can be used to determine MDC, also reported as the minimum difference to be considered real or smallest real difference [45,46].

The overall scale and all subscales of the C-RC-QOL showed no ceiling or floor effects. The experts’ assessment confirmed that the items of the C-RC-QOL were relevant for the construct to be measured and for the patients with RC disorders. Although some responses were missing from the symptoms and physical complaints and the work-related subscales, the missing rate was low (<5%) and was unlikely to have been caused by the patients' not being able to understand the scale. It was more likely a result of idiosyncratic responses from individual patients. In summary, we concluded that the C-RC-QOL had good content validity.

The correlations between the C-RC-QOL and the SF-36 and OSS were consistent with our hypotheses, indicating the good construct validity of the C-RC-QOL. This result is consistent with conclusions obtained in related studies [11,13,20,43]. The C-RC-QOL was most strongly correlated with the OSS. The OSS is a shoulder joint-specific measure but includes questions that are similar to some items on the C-RC-QOL. The SF-36’s physical subscales also had a high correlation with the C-RC-QOL, although the correlation was weaker than that between the OSS and the C-RC-QOL. The SF-36, being a generic measure would not be expected to correlate as well as a joint- or disease-specific measure with respect to the functional status of patients with RC disorders [47]. In addition, there was poor correlation between the mental subscales and physical subscales of the C-RC-QOL and the SF-36, which was logically reasonable and consistent with findings in other studies [11,21].

A scale’s responsiveness is an important factor in determining whether it can be used in prospective clinical research. Our study found that the overall scale and subscales of C-RC-QOL had good responsiveness. They were sensitive to changes in the functional status of patients who had received shoulder arthroscopic surgery. The values of ES and SRM in the present study were slightly larger than those in other studies [11, 48]. This is possibly explained by the fact that the treatment our patients received was arthroscopic surgery, whereas other studies included both surgery and conservative treatment, resulting in different degrees of improvement in functional status.

In addition, the unidimensionality of the five subscales of the C-RC-QOL was respected according to PCA of the residuals. Unidimensionality is a core assumption of item response theory models. In fact, it ensures that the person’s response to each item that measures the analyzed construct is accounted for by his/her amount of that trait, and not by other factors [49]. In addition, the unidimensionality of the RC-QOL has not been analyzed before.

## 5. Conclusion

In summary, we successfully developed a Chinese version of the RC-QOL and confirmed that this version was easy to use and had good reliability, validity and responsiveness. It may be used to assess the functional status of Chinese patients with RC disorders in clinical studies or treatment, helping doctors or researchers collect necessary required data.

## Supporting information

S1 Text (PDF)

S2 Text (PDF)

S1 Table (DOCX)

S2 Table (XLSX)
