# Supplementary material for: Reliability, validity and responsiveness of the Chinese version of the Rotator Cuff Quality of Life Index (RC-QOL) in patients with rotator cuff disorders
Source: PLoS One. 2018 Nov 8;13(11):e0206347. doi: 10.1371/journal.pone.0206347 (PMC6224054; doi:10.1371/journal.pone.0206347)
Supplement: S1 Text — (PDF) [file pone.0206347.s001.pdf]

Quality-of Life Assessment in Rotator Cuff Patients

Section A symptoms and physical complaints

The first section is related to symptoms and physical complaints

1. With any prolonged activity (ie. greater than half an hour), how much pain or discomfort do you experience in your shoulder?

0 ◆————————————————◆ 100

Severe pain No pain at all

2. With respect to your overall shoulder function, how much are you troubled by stiffness or loss of motion?

0 ◆————————————————◆ 100

Severe troubled Not troubled at all

3. With respect to your overall shoulder function and considering the strength of your muscles, how weak is your shoulder?

0 ◆————————————————◆ 100

Totally weak Not weak at all

4. With respect to bathing or taking a shower, how much pain/difficulty do you experience because of your shoulder?

0 ◆————————————————◆ 100

Severe pain/difficulty No pain/difficulty at all

5. With respect to putting on or removing clothing over your head, how much pain/difficulty do you experience because of your shoulder?

0 ◆————————————————◆ 100

Severe pain/difficulty No pain/difficulty at all

6. With respect to putting on a belt through the loop holes of a pair of pants that you are wearing, how much pain/difficulty do you experience because of your shoulder?

0 ◆————————————————◆ 100

Severe pain/difficulty No pain/difficulty at all

7. With respect to cutting food for preparation or at meals, how much pain/difficulty do you experience because of your shoulder?

0 ◆————————————————◆ 100

Severe pain/difficulty No pain/difficulty at all

8. With respect to doing household chores (ie. mopping floor/vacuuming the rug, ironing clthes, making a bed, scrubbing pots/pans, cleaning bathtub/toilet), how much pain/difficulty do you

experience because of your shoulder?

0 ◆————————————————◆ 100

Severe pain/difficulty No pain/difficulty at all

9. With respect to carrying 4.5 to 6.8 kg (10 to 15 lb), with your arm at your side (ie. carrying a heavy briefcase, small suitcase, or shopping bags), how much pain/difficulty do you experience because of your shoulder?

0 ◆————————————————◆ 100

Severe pain/difficulty No pain/difficulty at all

10. With respect to cutting the grass, raking the lawn, or shoveling snow, how much pain/difficulty do you experience because of your shoulder?

0 ◆————————————————◆ 100

Severe pain/difficulty No pain/difficulty at all

11. Do you have pain/difficulty falling asleep because of your shoulder?

0 ◆————————————————◆ 100

Severe pain/difficulty No pain/difficulty at all

12. Are you awakened from sleep because of your shoulder?

0 ◆————————————————◆ 100

Always awakened Never awakened

13. With respect to driving a motor vehicle, how much pain/difficulty do you experience because of your shoulder?

0 ◆————————————————◆ 100

Severe pain/difficulty No pain/difficulty at all

14. With respect to opening and closing a door with your affected arm, how much pain/difficulty do you experience because of your shoulder?

0 ◆————————————————◆ 100

Severe pain/difficulty No pain/difficulty at all

15. With respect to reaching (ie. into the back of car) with your affected arm, how much pain/difficulty do you experience because of your shoulder?

0 ◆————————————————◆ 100

Severe pain/difficulty No pain/difficulty at all

16. Indicate the point ranging from 0 to 100 which most closely describes your overall present level of shoulder pain.

0 ◆————————————————◆ 100

Severe pain/difficulty No pain/difficulty at all

A there any other physical issues that you feel should be addressed?

Section B   work-related concerns

The following questions are related to your job or vocation (ie. work-related concerns). The questions are regarding your ability to function at work and the extent to which your shoulder has affected your current work-related concerns. If you are a full-time student/homemaker, then consider this and any part-time work together. Consider the last 3 months.

If you are not working for reasons other than your shoulder problem, proceed to question 21

Indicate, with a slash on the line, the point ranging from 0 to 100 that most closely represents your situation.

17. With respect to working with your arm at shoulder level, how much pain/difficulty do you experience because of your shoulder?

0 ◆————————————————————◆ 100

Severe pain/difficulty   No pain/difficulty at all

18. With respect to working with your arm above shoulder level, how much pain/difficulty do you experience because of your shoulder?

0 ◆————————————————————◆ 100

Severe pain/difficulty   No pain/difficulty at all

19. How much of the time are you concerned with missing days from work because of problems with or re-injury to your shoulder? (Make a slash at the extreme left if you are unable to work because of your shoulder)

0 ◆————————————————————◆ 100

Greatly concerned   Not concerned at all

20. How much of the time are you concerned that the activities you do at work may result in the state of your shoulder becoming worse? (Make a slash at the extreme left if you are unable to work because of your shoulder)

0 ◆————————————————————◆ 100

All of the time   None of the time

Are there any other occupational issues that you feel should be addressed?

Section C   recreational activities and sport participation or competition.

The following questions are being asked with respect to your recreational activities and sport participation or competition. The questions are concerned with the extent to which your painful shoulder affects your ability to function and participate in these activities. Consider the last 3

months.

If you are not involved in any sporting activities whatsoever, proceed to question 25.

21. With respect to participating in general sports activities, how much pain/difficulty do you experience because of your shoulder?

0 ◆────────────────────◆ 100

Severe pain/difficulty No pain/difficulty at all

22. With respect to participating in upper-extremity sports (eg. baseball, tennis, golf, squash, volleyball, swimming, throwing), how much pain/difficulty do you experience because of your shoulder?

0 ◆────────────────────◆ 100

Severe pain/difficulty No pain/difficulty at all

23. How much of the time are you concerned that your sporting/recreational activities may result in the status of your shoulder becoming worse?

0 ◆────────────────────◆ 100

All of the time None of the time

24. With respect to your current level of athletic or recreational performance, how does it compare with your pre-injury level?

0 ◆────────────────────◆ 100

Totally limited No limitations

Section D lifestyle

The following questions are being asked with respect to your lifestyle. The questions are concerned with your lifestyle in general and the extent to which your painful shoulder affects activities other than those related to your work and sports/recreation. Consider the last 3 months.

25. How often do you have to concern yourself with general safety (eg. carrying small children, working in the yard, climbing a ladder, using power tools) with respect to your injured shoulder?

0 ◆────────────────────◆ 100

All of the time None of the time

26. How much has your enjoyment of life been limited by your shoulder problem?

0 ◆────────────────────◆ 100

Totally limited No limitations

27. How often are you aware of your shoulder problem?

0 ◆────────────────────◆ 100

All of the time None of the time

28. With respect to your lifestyle as it relates to you and your family together, how often are you concerned about your shoulder?

0 ◆―――――――――――――――――――――◆ 100

All of the time None of the time

29. You have had your problem shoulder for some time. During this time, have you modified your lifestyle to avoid potentially damaging activities to your shoulder?

0 ◆―――――――――――――――――――――◆ 100

Totally modified No modifications

Section E social and emotional aspects

The following questions are regarding the social and emotional aspects of your shoulder problem. The questions are concerned with your attitudes and feelings as they relate to your painful shoulder. Consider the last 3 months.

30. Do you experience difficulty making decisions at home or at work because of your shoulder problem?

0 ◆―――――――――――――――――――――◆ 100

Extremely difficult Not difficult at all

31. Do you have peace of mind, or are you too worried to sleep at night because of your shoulder problem?

0 ◆―――――――――――――――――――――◆ 100

Extremely worried Not worried at all

32. Are you afraid of reinjuring your shoulder?

0 ◆―――――――――――――――――――――◆ 100

Extremely afraid Not afraid at all

33. Do you experience psychological difficulty when engaging in sexual activity because of your shoulder problem?

0 ◆―――――――――――――――――――――◆ 100

Extremely difficult Not difficult at all

34. Does your shoulder problem interfere with your ability to socialize with friends and family?

0 ◆―――――――――――――――――――――◆ 100

Unable to socialize Able to socialize fully
