# Supplementary material for: Reliability, validity and responsiveness of the Chinese version of the Rotator Cuff Quality of Life Index (RC-QOL) in patients with rotator cuff disorders
Source: PLoS One. 2018 Nov 8;13(11):e0206347. doi: 10.1371/journal.pone.0206347 (PMC6224054; doi:10.1371/journal.pone.0206347)
Supplement: S2 Text — (PDF) [file pone.0206347.s002.pdf]

中国肩袖患者生命质量评估量表（C-RC-QOL）

第一部分　症状和体征

该部分与您的症状和体征相关，在线上划出一个斜线以表示您对每道题的回答打分。

1. 当您的肩关节进行长时间的活动后（例如超过半小时），您肩膀感受到的疼痛有多严重？

0 ◆——————————————◆ 100

非常痛　　　　　　　　　　　　　　一点也不痛

2. 关于您肩膀的整体功能，肩膀的僵硬或活动受限有多困扰您？

0 ◆——————————————◆ 100

非常困扰　　　　　　　　　　　　　　一点也困扰

3. 关于您肩膀的整体功能以及肌肉的力量，您认为您的肩膀状态有多弱？

0 ◆——————————————◆ 100

非常弱　　　　　　　　　　　　　　一点也不弱

4. 在您洗澡或淋浴的时候，您感觉您肩膀的疼痛或活动困难有多严重？

0 ◆——————————————◆ 100

非常痛/困难　　　　　　　　　　　　　　一点也不痛/困难

5. 在您穿上或脱下一些圆领毛衣或 T 恤衫时，您肩膀的疼痛或活动困难有多严重？

0 ◆——————————————◆ 100

非常痛/困难　　　　　　　　　　　　　　一点也不痛/困难

6. 当您将皮带穿戴到裤子上时，您肩膀的疼痛或活动困难有多严重？

0 ◆——————————————◆ 100

非常痛/困难　　　　　　　　　　　　　　一点也不痛/困难

7. 当您使用双手拿起碗筷吃饭时，您肩膀的疼痛或活动困难有多严重？

0 ◆——————————————◆ 100

非常痛/困难　　　　　　　　　　　　　　一点也不痛/困难

8. 当您做家务时（例如拖地、熨烫衣物、铺床、擦洗锅盆、清洁浴缸厕所等），您肩膀的疼痛或活动困难有多严重？

0 ◆——————————————◆ 100

非常痛/困难　　　　　　　　　　　　　　一点也不痛/困难

9. 当您在单手搬运一个 9 斤到 14 斤重的物品时（例如单手搬动公文包、小行李箱或购物袋时），您搬运物品一侧的肩膀疼痛或活动困难有多严重？

0 ◆——————————————◆ 100

非常痛/困难　　　　　　　　　　　　　　一点也不痛/困难

10. 当您在割草、耙草或铲雪时，您肩膀的疼痛或活动困难有多严重？

0 ◆————————————————◆ 100

非常痛/困难　　　　　　　　　　　　　　　一点也不痛/困难

11. 当您睡觉时您肩膀的疼痛有多严重？

0 ◆————————————————◆ 100

非常痛　　　　　　　　　　　　　　　　　一点也不痛

12. 您曾因您的肩膀疼痛而半夜醒来吗？

0 ◆————————————————◆ 100

总是因此醒来　　　　　　　　　　　　　　从来没因此醒来

13. 当您驾驶摩托或骑自行车时，您肩膀的疼痛或活动困难有多严重？

0 ◆————————————————◆ 100

非常痛/困难　　　　　　　　　　　　　　　一点也不痛/困难

14. 当您使用患肢打开或关闭一个门时，您活动一侧肩膀的疼痛或活动困难有多严重？

0 ◆————————————————◆ 100

非常痛/困难　　　　　　　　　　　　　　　一点也不痛/困难

15. 当您使用患肢到达某个地方时（例如进入车中），您活动一侧肩膀的疼痛或活动困难有多严重？

0 ◆————————————————◆ 100

非常痛/困难　　　　　　　　　　　　　　　一点也不痛/困难

16. 您觉得使用 0-100 分来整体评估您肩膀疼痛的水平，最恰当的分数是多少？

0 ◆————————————————◆ 100

非常痛/困难　　　　　　　　　　　　　　　一点也不痛/困难

您还有其它一些感到的肩关节功能或体征问题需要说明的吗？

第二部分　与工作有关的问题

以下的问题与您的工作或职业有关。 这些问题是评估您在工作中的肩关节功能状态以及您的肩膀症状对当前工作的影响程度。如果您是全日制学生或家庭主妇，请将这些情况代入到您平时的兼职工作中考虑。您仅只需根据近 3 个月内的情况来回答下列问题。

如果您的肩膀症状或体征没有影响到您的工作，请直接跳到第 21 题进行作答。

注意，在线上划出一个斜线以表示您对每道题的回答打分。

17. 当您工作时上臂处于您肩膀同样水平时，您肩膀的疼痛或活动困难有多严重？

0 ◆————————————————◆ 100

非常痛/困难　　　　　　　　　　　　　　　一点也不痛/困难

18. 当您工作时上臂超过您肩膀的高度时，您肩膀的疼痛或活动困难有多严重？

0 ◆——————————————————◆ 100

非常痛/困难　　　　　　　　　　　　一点也不痛/困难

19. 平时您是否经常担心以后可能因为肩膀的问题导致您不能继续参加工作？（如果你因肩膀的问题而完全无法工作，请在最左侧划上斜线）

0 ◆——————————————————◆ 100

随时担心　　　　　　　　　　　　　从来不担心

20. 平时您是否经常担心工作可能导致您肩膀的症状或体征日益严重？（如果你因肩膀的问题而完全无法工作，请在最左侧划上斜线）

0 ◆——————————————————◆ 100

随时担心　　　　　　　　　　　　　从来不担心

您还有其它与工作相关的问题需要特别说明的吗？

第三部分　娱乐活动以及日常运动（或比赛）

以下这些问题主要是关注您肩膀的问题对于您日常娱乐活动、运动（或比赛）的影响。这些问题关注因肩膀的疼痛、功能障碍等影响您参与上述活动的能力。您仅只需根据近 3 个月内的情况来回答下列问题。

如果您的肩膀症状或体征没有影响到您的参与上述活动，请直接跳到第 25 题进行作答。

21. 当您参加日常一般的体育活动时，您肩膀的疼痛或活动障碍有多严重？

0 ◆——————————————————◆ 100

非常痛/困难　　　　　　　　　　　　一点也不痛/困难

22. 当您参加主要使用上肢的相关体育活动时（例如棒球、网球、高尔夫球、排球、壁球、游泳、投掷等），您肩膀的疼痛或活动障碍有多严重？

0 ◆——————————————————◆ 100

非常痛/困难　　　　　　　　　　　　一点也不痛/困难

23. 平时您是否经常担心参与日常运动可能会导致您肩膀的症状或体征日益严重？

0 ◆——————————————————◆ 100

随时担心　　　　　　　　　　　　　从来不担心

24. 基于您目前参与娱乐活动或运动的表现水平，相较于您肩关节出现症状之前的水平如何？

0 ◆——————————————————◆ 100

差很远　　　　　　　　　　　　　　没什么变化

第四部分　日常生活

下列问题是关于您日常生活方面。这些问题主要关注您肩膀症状或体征对于您日常生活中的一些活动（除开工作、运动、娱乐活动等）的影响程度。您同样仅只需根据近 3 个月内的情况来回答下列问题。

25. 您是否经常担心肩膀的问题可能影响到您自身的日常生活安全？（例如携带小孩、在院子里工作、攀爬梯子、使用电动工具等）

0 ◆————◆ 100

随时担心　　　　从来不担心

26. 您肩膀的问题对于您享受日常生活的影响有多大？

0 ◆————◆ 100

非常大　　　　没什么影响

27. 日常生活中您是否经常意识到自己肩膀存在问题？

0 ◆————◆ 100

随时　　　　从来没有

28. 在日常生活中，当您与家人共处时，是否经常意识到自己肩膀存在问题？

0 ◆————◆ 100

随时　　　　从来没有

29. 你肩膀的问题已经存在一段时间了，而在这段时间里，你是否为避免一些潜在因素伤害到您的肩膀而改变了一些生活方式？

0 ◆————◆ 100

完全改变了　　　　没有任何改变

第五部分　社会和心理方面

下列问题关注您肩膀的症状及体征对您社会及心理方面的影响，主要评估肩膀的疼痛对于您日常生活态度及感受的影响。您同样仅只需根据近 3 个月内的情况来回答下列问题。

30. 您是否由于您肩膀的问题在家庭生活或工作中遇到困难？

0 ◆————◆ 100

非常困难　　　　没有困难

31. 您肩膀的问题是否导致您夜晚失眠？

0 ◆————◆ 100

每天晚上　　　　从来没有

32. 您害怕您的肩膀再次受到创伤吗？

0 ◆————◆ 100

非常害怕　　　　不害怕

33. 您肩膀的问题是否影响到了您日常的性生活？

0 ◆————————————————◆ 100

不能进行性生活　　　　　　　　　　　　　　　没有影响

34. 您肩膀的问题是否影响了您与朋友及家人的相处？

0 ◆————————————————◆ 100

不能正常相处　　　　　　　　　　　　　　　　没有影响
